# Effectiveness, tolerability, and retention of the ketogenic diet for infantile epileptic spasms syndrome: a single-center cohort study

**DOI:** 10.3389/fneur.2026.1701141

**Published:** 2026-02-25

**Authors:** Fen Zhao, Wandong Hu, Wenchao Zhang, Yi Lu, Juan Li, Hongwei Zhang

**Affiliations:** 1 Department of Neurology, Children’s Hospital Affiliated to Shandong University, Jinan Children’s Hospital, Jinan, China; 2 Department of Radiology, Children’s Hospital Affiliated to Shandong University, Jinan Children’s Hospital, Jinan, China

**Keywords:** effectiveness, infantile epileptic spasms syndrome, ketogenic diet, retention, safety

## Abstract

**Objective:**

This study aimed to investigate the effectiveness, tolerability, and retention of the ketogenic diet (KD) in patients with infantile epileptic spasms syndrome (IESS).

**Methods:**

In this single-center prospective cohort study, baseline data were collected from the Children’s Hospital Affiliated to Shandong University. Follow-up assessments were conducted at 3, 6, and 12 months after initiating KD. Outcomes included seizure frequency, adverse reactions, and retention rates. Survival analysis was performed to examine the association between retention rates and follow-up duration.

**Results:**

A total of 74 patients with IESS were admitted. The seizure response rates were 56.8% (42/74), 41.9% (31/74), and 25.7% (19/74) at 3, 6, and 12 months, respectively. The corresponding seizure-free rates were 13.5% (10/74), 13.5% (10/74), and 8.1% (8/74), respectively. Retention rates at 3, 6, and 12 months were 70.2% (52/74), 43.2% (32/74), and 25.7% (19/74), respectively. Survival analysis indicated that retention rates decreased over time, most markedly within the first 3 months. Responders to the KD exhibited significantly higher retention rates than non-responders throughout the 12 months (HR = 0.35, 95% CI: 0.19–0.64; *p* < 0.001). Adverse reactions were reported in 20.3% of patients, with gastrointestinal symptoms being the most common (16.2%), including constipation (6.8%), diarrhea (5.4%), and vomiting (4.0%).

**Conclusion:**

The KD demonstrated favorable effectiveness and an acceptable safety profile in patients with IESS, particularly in those who did not respond to first-line antiseizure medications (ASMs). The higher retention rates among responders supports its utility in children with IESS. Therefore, early initiation of the KD should be considered for IESS patients who do not respond to first-line ASMs.

## Introduction

Infantile epileptic spasm syndrome (IESS), previously referred to as infantile spasms or West syndrome, was formally defined by the International League Against Epilepsy (ILAE) in 2022 (1). This severe developmental epileptic encephalopathy presents in infancy or early childhood and is characterized by epileptic spasms, hypsarrhythmia observed on an electroencephalogram (EEG), or developmental regression, although not all features are necessarily present before the onset of spasms (1). The incidence of IESS ranges from 2 to 5 per 10,000 live-born infants, accounting for approximately 10% of all epilepsies in children under the age of 3 years (2). Evidence suggests that the higher the geographical latitude, the higher the incidence rate (3). It typically occurs between 3 and 12 months of age, with a peak incidence between 4 and 6 months (4). Many affected children experience poor long-term prognosis, presenting moderate to severe intellectual disability and drug-resistant epilepsy (DRE). This highlights the critical importance of early intervention in improving neurological prognosis.

Current treatment approaches are various. The American Academy of Neurology (AAN) recommends low-dose adrenocorticotropic hormone (ACTH) as a first-line therapy for IESS regardless of the underlying cause (5). In Japan, treatment strategies are more etiology-specific and may include synthetic ACTH, pyridoxine, or valproate (6). In the UK, vigabatrin (VGB) is the most common first-line treatment (6). In non-pharmacological therapies, surgery is only suitable for patients with a well-defined epileptogenic focus, and neuromodulation has been demonstrated to have limited efficacy in those who are unresponsive to antiseizure medications (ASMs) (6). Despite undergoing the above standard treatments, a considerable number of patients still experience continuous, frequent seizures and progressive developmental impairment (7), indicating an urgent need for novel therapeutic strategies to improve outcomes in IESS.

The ketogenic diet (KD) is a nutritional approach that is high in fat, provide adequate protein, and is very low in carbohydrates. This diet induces a metabolic shift that promotes the use of ketone bodies as an alternative energy source (7). Initially introduced as a treatment for epilepsy, its use declined after the introduction of antiseizure medications (ASMs) in 1938 (7). Since the mid-1990s, the KD has regained prominence as an alternative therapy for DRE in children, particularly due to its potential cognitive benefits. Compared to surgical options, the use of KD is reversible, inexpensive, and widely accessible. At present, it is well-established that KD is both effective and safe in the treatment of DRE (7–10). However, clinical data on KD treatment for IESS are limited, particularly regarding longitudinal retention rates.

Therefore, this single-center prospective cohort study aims to explore the effectiveness, tolerability, and retention rates of KD in patients with IESS, with the goal of providing further evidence and guidance for clinical practices.

## Methods

### Study design

This was a single-center prospective cohort study. The study was conducted involving patients with IESS who were treated with the KD at the Children’s Hospital Affiliated to Shandong University between July 2024 and July 2025. This study was approved by the Ethics Committee of Children’s Hospital Affiliated to Shandong University (No. SDFE-IRB/P-2023016). All family members of patients agreed to participate in this study and provided signed informed consent.

Baseline data were collected before the initiation of the KD, and the patients were subsequently followed up after starting the diet. These baseline data included information on sex, age of onset, seizure type, seizure frequency, epilepsy syndrome, etiology, and the use of ASMs. Follow-up assessments were performed at 3, 6, and 12 months after initiating KD through outpatient visits and telephone interviews. Data collected during the follow-up included adherence to the KD, seizure types and frequency, and any observed adverse effects. During this treatment, a nutritionist continuously monitored and recorded patients’ compliance and any adverse reactions. The seizure frequency was recorded by the patients or caregivers in the epilepsy seizure diaries that we provided.

### Patients

The inclusion criteria for patients were as follows: (1) aged between 0 and 18 years, (2) diagnosed with IESS based on the updated guidelines proposed by the International League Against Epilepsy (ILAE) in 2025 (11), and (3) a follow-up period of at least 3 months. The exclusion criteria included patients with a metabolic profile showing contraindications to the KD and those who were lost to follow-up or who had incomplete data.

According to the previous literature review, the effective rate of KD treatment ranged between 50 and 75% in the 3-month follow-up (12, 13). We calculated that the minimum sample size required was 37 cases using PASS software. Therefore, 74 patients in our study met the estimated sample size.

### Dietary protocol

Patients underwent a comprehensive pre-diet evaluation by clinicians and nutritionists at least 7 days before initiating the KD. Baseline assessments included weight and height, blood biochemical analyses (serum lipids and albumin), and renal ultrasonography to exclude contraindications to KD. The choice of dietary therapy—either classical KD or modified Atkins diet (MAD)—and its specific ratio was determined by the attending physician. For patients on classical KD, the initial lipid-to-non-lipid ratio ranged from 0.8:1 to 4:1, and ensuring the ratio in the maintenance period was between 2:1 and 4:1, whereas those on MAD received a ratio of approximately 1.5:1 in accordance with the Johns Hopkins protocol. Throughout the dietary intervention, metabolic parameters, including blood glucose, blood β-hydroxybutyrate (BHB), and urinary ketones, were regularly monitored. Adverse reactions were documented, and the KD regimen was adjusted as necessary. The maintenance period was continued for at least 1 month to evaluate effectiveness. The final follow-up was conducted in July 2025. Baseline ASMs remained unchanged during the KD treatment period.

### Outcomes

The primary outcome of the study was the seizure response rate, defined as the proportion of patients achieving a greater than 50% reduction in seizure frequency compared to baseline, assessed at 3, 6, and 12 months after the initiation of the KD. Secondary outcomes included seizure-free rates (defined as the proportion of patients experiencing complete cessation of seizures) at the same follow-up intervals, along with subgroup analyses of response rates for different etiologies and seizure types. Additional outcomes comprised retention rates (defined as the proportion of patients still adhering to the KD at their last follow-up) stratified by responders and non-responders, as well as documentation of adverse events, which were evaluated based on parental reports and clinical observations.

### Statistical analysis

Patients were categorized into responder and non-responder groups based on whether they achieved a reduction of more than 50% in seizure frequency at the 3-month follow-up. The baseline and clinical characteristics of both groups and those of the total population were summarized and compared using standard descriptive statistical methods.

Quantitative data were expressed as means (standard deviations) for normally distributed variables, and as medians and interquartile ranges for non-normally distributed variables. Categorical and ordinal data were summarized as counts and percentages, with group comparisons performed using the chi-squared test or Fisher’s exact test, as appropriate. Retention in the KD was evaluated over the entire follow-up period using survival analysis. The results are presented as hazard ratios (HRs) with their corresponding 95% confidence intervals (95% CIs).

All statistical analyses in this study were performed using SPSS Statistics (version 26). A *p*-value of <0.05 was considered to indicate statistical significance.

## Results

### Baseline data

A total of 85 patients with IESS were initially enrolled and received KD therapy. After excluding 11 patients due to incomplete data or loss of follow-up, 74 patients were included in the final analysis. There were 47 male (63.5%) and 27 female patients (36.5%). The median age at initiation of the KD was 1.1 years, with a median epilepsy duration of 5.45 months. Before the initiation of the KD, the median number of ASMs at baseline was 3. The median baseline seizure frequency was four times per day.

Regarding seizure types, 12.2% (9/74) of patients exhibited focal spasms only, 73.0% (54/74) presented with generalized seizures only, and 14.8% (11/74) experienced both seizure types. Etiological classification revealed genetic etiology in 31.1% (23/74), structural etiology in 27.0% (20/74), and unknown etiology in 41.9% (31/74) of patients. None of these baseline variables showed statistically significant differences between responders and non-responders to KD therapy (Table 1).

**Table 1:** General information of patients and comparison of the effective rate of KD in different variables at the 3-month follow-up.

Index	Total (*n* = 74)	Responders (*n* = 42)	Non-responders (*n* = 32)	*p*-value
Age of seizure onset (months)	6.00 (3.30, 12.01)	6.00 (3.67, 12.98)	5.75 (2.13, 11.36)	>0.05
Sex				>0.05
Male	47 (63.5%)	29 (61.7%)	18 (38.3%)	
Female	27 (36.5%)	13 (48.1%)	14 (51.9%)	
Onset age of KD (year)	1.1 (0.78, 2.01)	1.13 (0.75, 2.36)	1.09 (0.79, 1.71)	>0.05
Duration of epilepsy (months)	5.45 (2.47, 12.78)	5.35 (1.90, 12.78)	5.75 (2.78, 15.60)	>0.05
Seizure frequency at baseline (daily)	4.00 (3.00, 10.00)	3.00 (3.00, 6.50)	5.00 (3.00, 20.00)	0.05
Seizure type				>0.05
Focal	9(12.2%)	5 (55.6%)	4 (44.4%)	
Generalized	54(73.0%)	31 (57.4%)	23 (42.6%)	
Both	11(14.8%)	6 (54.5%)	5 (45.6%)	
Etiology				>0.05
Genetic	23(31.1%)	9 (39.1%)	14 (60.9%)	
Structural	20(27.0%)	13 (65.0%)	7 (40.0%)	
Unknown	31(41.9%)	20 (64.5%)	11 (35.5%)	
Hypsarrhythmia in EEG	24 (32.4%)	14 (58.3%)	10 (41.7%)	>0.05
The number of ASMs before KD	3.00 (2.00, 3.00)	3.00 (2.00, 3.00)	3.00 (2.00, 3.00)	>0.05

KD, ketogenic diet; EEG, electroencephalogram; ASMs, anti-seizure medication.

Regarding dietary therapy, 50 patients were treated with classical KD, and 24 patients were treated with MAD. In comparison between these two dietary treatments, none of the baseline variables or seizure response rates showed statistically significant differences. Among the 50 patients with classical KD during the maintenance phase, the distribution of KD ratios was as follows: 54% (27/50) received a 4:1 ratio, 26% (13/50) received a 3:1 ratio, and the remaining 20% (10/50) received a 2:1 ratio. No statistically significant difference in the distribution of these diet ratios was observed between responders and non-responders.

### Primary and secondary outcomes

**Figure 1:**
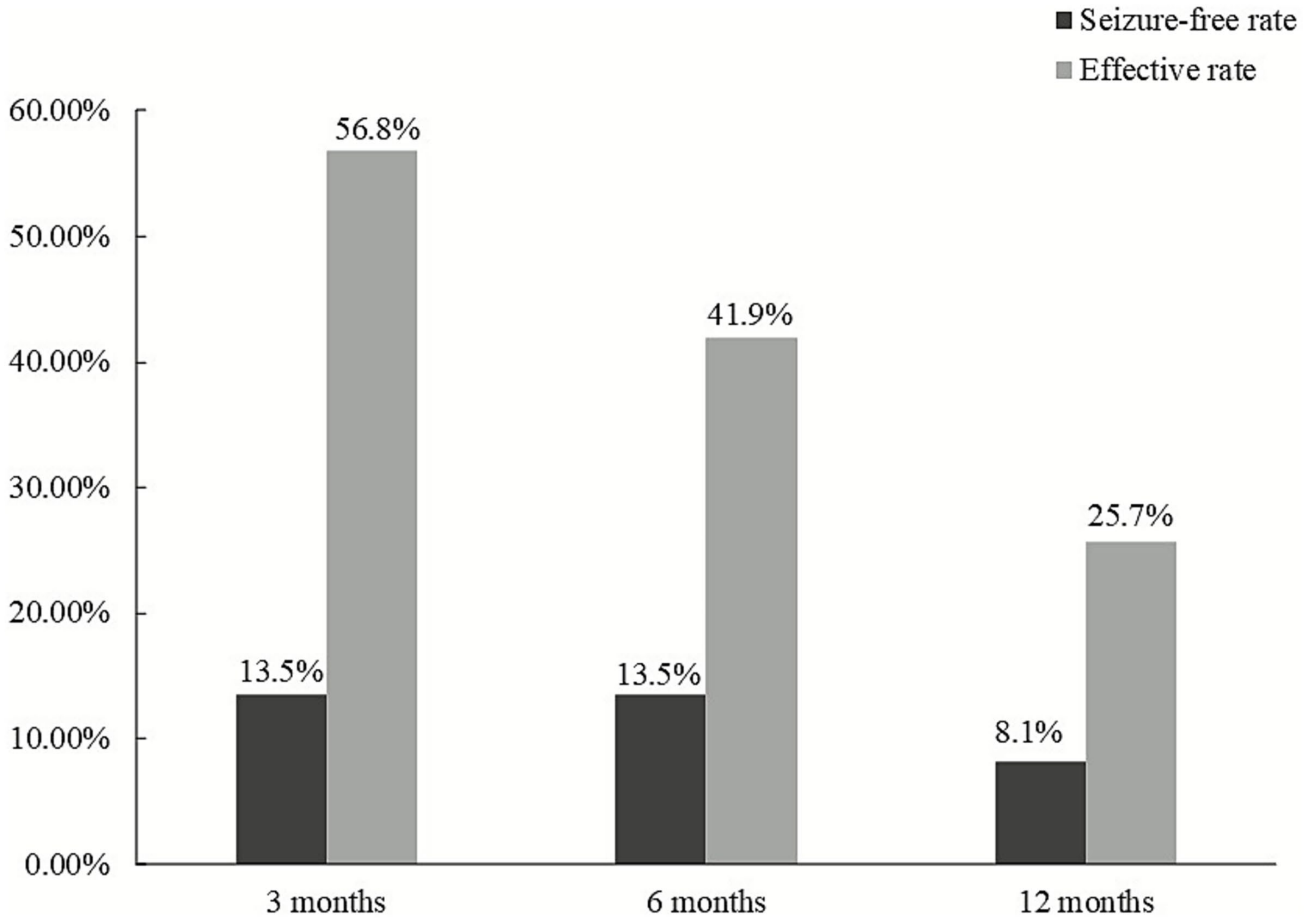
Overall seizure response rate and seizure-free rate at 3-, 6-, and 12-month follow-up.

The response rates to seizures were 56.8% (42/74), 41.9% (31/74), and 25.7% (19/74) at 3-, 6-, and 12-month follow-ups, respectively (Figure 1). The seizure-free rates were 13.5% (10/74), 13.5% (10/74), and 8.1% (6/74) at 3-, 6-, and 12-month follow-ups, respectively (Figure 1).

**Figure 2:**
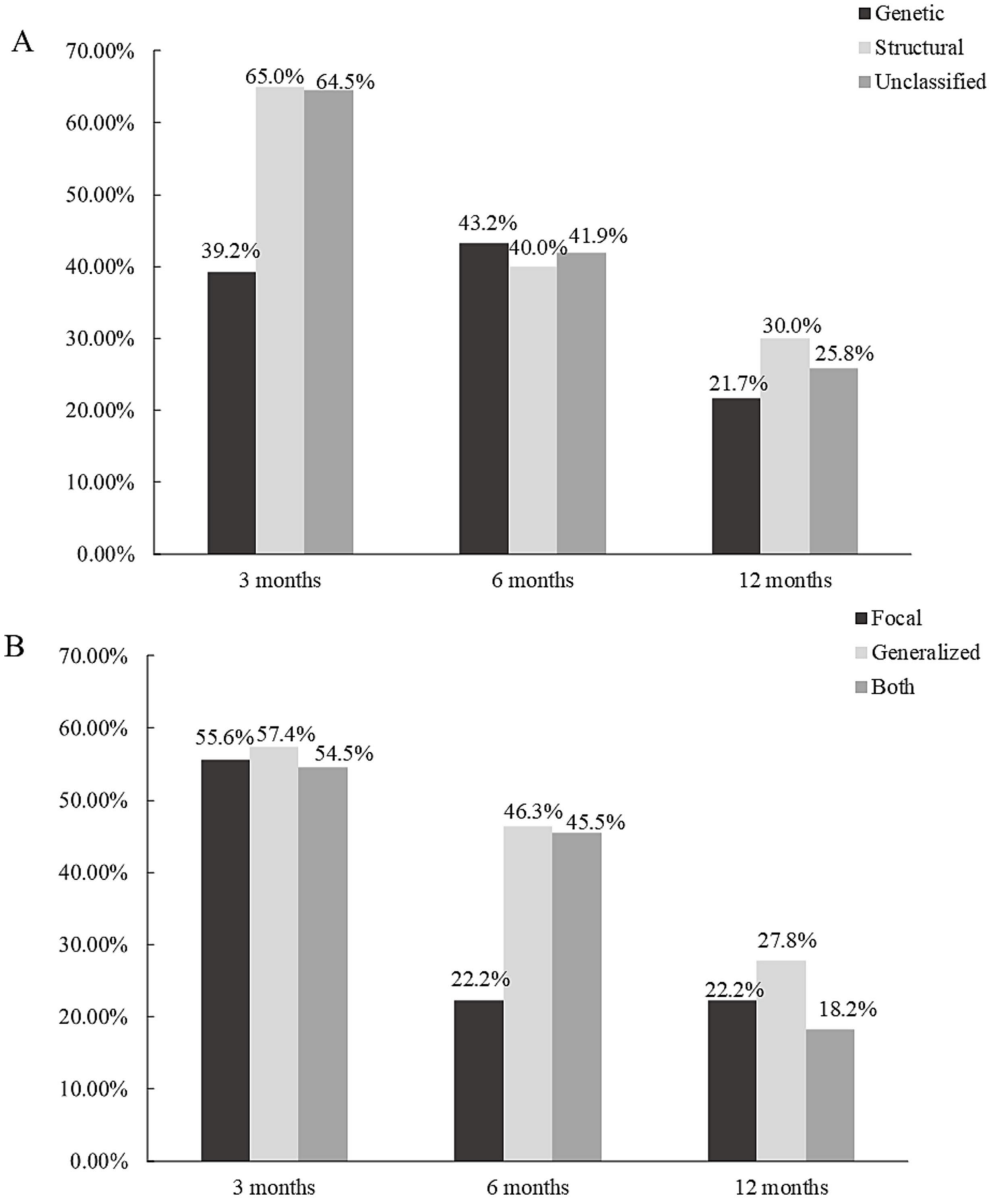
**(A)** Seizure response rate of different etiologies at 3-, 6-, and 12-month follow-up. **(B)** Seizure response rate of different seizure types at 3-, 6-, and 12-month follow-up.

When examining the different etiological subgroups, the seizure response rates for structural and unknown etiologies were broadly comparable at each follow-up period and exhibited a declining trend over time. Both subgroups demonstrated response rates exceeding 60% at 3 months, approximately 40% at 6 months, and approximately 25% at 12 months (Figure 2A). In the genetic etiology subgroup, the seizure response rates were 39.2% (29/74), 43.2% (32/74), and 21.7% (16/74) at the 3-, 6-, and 12-month follow-ups, respectively (Figure 2A). No statistically significant differences in response rates were observed among the three etiological subgroups.

In terms of seizure type subgroups, the seizure response rate of focal spasms was 55.6% (5/9) at 3-month follow-up, which was higher than 22.2% (2/9) at 6-month follow-up and 12-month follow-up. For patients with generalized seizures and those with both seizure types, the seizure response rates were broadly comparable at 3-month and 6-month follow-ups, with rates of approximately 55 and 45%, respectively. Both groups also experienced a decline in response rates over time (Figure 2B). No statistically significant differences in response rates were observed among the three seizure-type categories.

### Retention rate

**Figure 3:**
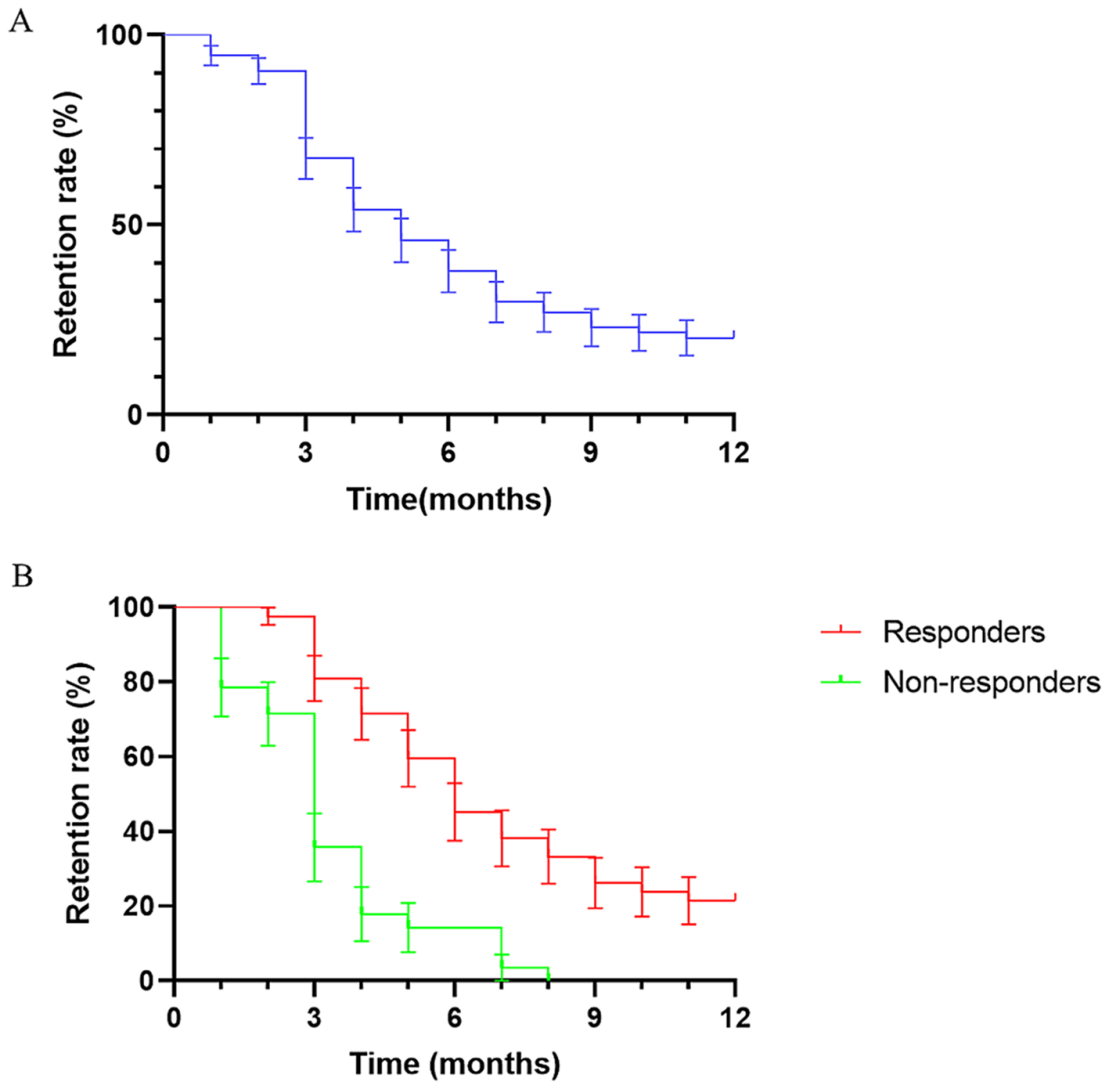
**(A)** Survival curve of the overall retention rate over follow-up time. **(B)** Survival curve of the retention rate in the response and non-response groups over follow-up time.

The final follow-up was conducted 12 months after KD initiation. Retention rates at 3, 6, and 12 months were 70.2% (52/74), 43.2% (32/74), and 25.7% (19/74), respectively. Significant differences in retention rates were observed across time points (*p* < 0.001), with pairwise comparisons reaching statistical significance (*p* < 0.05 for each pair). Survival analysis showed a progressive decline in KD retention over time, with the most pronounced decrease occurring within the first 3 months (Figure 3A).

Responders exhibited a median retention time of 6 months, while non-responders had a median retention time of 3 months among. Survival analysis demonstrated significantly higher retention rates among responders throughout the 12-month follow-up period (HR = 0.35, 95% CI: 0.19–0.64; *p* < 0.001) (Figure 3B).

### Adverse reactions

Overall, 15 patients (20.3%) experienced adverse reactions during KD therapy. The most common adverse reactions involved the gastrointestinal system (*n* = 12, 16.2%), including constipation (*n* = 5, 6.8%), diarrhea (*n* = 4, 5.4%), and vomiting (*n* = 3, 4.0%). These gastrointestinal adverse reactions tended to be mild and easily corrected with minimal interventions and did not lead to discontinuation of the diet. In addition, nephrolithiasis occurred in three patients but gradually disappeared through adjustments to their diet ratio by the nutritionist and increased water intake.

## Discussion

Our study adds to the growing body of evidence demonstrating the favorable effectiveness and safety of KD treatment in patients with IESS, particularly those who have proven refractory to first-line treatments such as ACTH and VGB. Furthermore, longitudinal retention decreased over time, most markedly within the first 3 months. The higher retention rate among responders supports its utility in children with IESS. Therefore, early initiation of KD should be considered for those with IESS unresponsive to first-line ASMs.

Our findings indicate a significant reduction in spasm frequency, with 56.8% of patients achieving a reduction of >50% at 3-month follow-up, which was consistent with the results of previous studies conducted in pediatric populations with IESS (14–17). A recent retrospective study involving 119 Chinese children with IESS showed that 47.9% exhibited effective seizure reduction after 16 weeks of KD treatment (18). The rapid onset of action observed in our responders within the first 2 weeks of diet initiation underscores the potential power of KD as a powerful acute intervention for breaking the harmful cycle of spasms and seizures, while also mitigating the associated developmental regression (17). Traditional clinical practice has initiated the KD following ACTH therapy (19, 20). However, a growing body of evidence now indicates that the initiation timing of KD is comparable to that of ACTH (21). A recent randomized controlled trial comparing the efficacy of KD and ACTH as first-line treatments showed that KD was not inferior to ACTH for the treatment of IESS. Furthermore, KD demonstrated fewer side effects and a lower rate of relapses compared to ACTH (12). A previous study indicated that potential epileptogenesis can occur during exceptionally long delays between the onset of spasms and the initiation of the KD and multiple pre-diet treatment failures were related to poor response rates of KD (22). Therefore, the above evidences emphasize the importance of early KD to improve the prognosis of patients.

In terms of different etiologies, we found better effectiveness in structural and unknown etiologies, with more than 60% at 3-month follow-up and 40% at 6-month follow-up. A systematic review also showed that IESS of unknown etiology seemed to have an increased probability of good efficacy (19). Regarding the structural etiology and KD, our previous study found that the seizure control effective rates were 52.4% at 3-month follow-up (23). Among the various structural etiologies, acquired brain injury and malformation of cortical development (MCD) were the most common structural abnormalities (23). Specifically, KD for IESS might involve multiple mechanism pathways, including altered neurotransmitter dynamics, neuroprotection through postictal hypoxia protection, and mitochondrial biogenesis. In the mechanism of KD for IESS with MCD, we speculated that it might be associated with the immature development of the cerebral cortex, leading to a preference for ketone bodies over glucose as an energy substrate (23). The immature cerebral cortex may better utilize ketone bodies (24). In the genetic etiology subgroup, our results indicated that the seizure response rates were 39.2% at the 3-month follow-up and 43.2% at the 6-month follow-up, which appear to be slightly lower than those in the subgroups with structural and unknown etiologies. However, a recent retrospective study from China has shown that 80% of patients with genetic etiology receiving the KD achieved a ≥75% reduction in seizures associated with IESS within the first 6 months of life (25). We speculate that the difference between our results and previous studies may be attributed to our small sample size and the possibility of undetected genetic abnormalities in the unknown etiology group.

Regarding safety, the adverse reactions observed were mild and manageable (26). Overall, 20.3% of patients in our study experienced adverse reactions during KD treatment. The most common adverse reactions were related to the gastrointestinal system (16.2%), including constipation, diarrhea, and vomiting. These gastrointestinal symptoms usually occurred in the initial few weeks of dietary therapy, which is consistent with previous literature (27). Among these symptoms, constipation is the most common adverse reaction during KD in infants with IESS, which was consistent with a previous study (28). In addition, long-term complications in children who have been on the KD for more than 2 years have not been reviewed systematically (26). In our study, nephrolithiasis occurred in three patients who were on KD therapy for more than 1 year; however, the condition gradually resolved with adjustments to the diet ratio by the nutritionist and increased water intake. However, a previous study reported instances of discontinuing the ketogenic diet due to severe nephrolithiasis (29). Crucially, no cases of serious or life-threatening complications were recorded. However, the high prevalence of these manageable side effects necessitates a proactive and vigilant multidisciplinary approach. Therefore, the comprehensive assessment of pre-diet complications and close monitoring for possible adverse reactions during the ketogenic diet are essential in infancy, a period of critical and rapid growth. Our data showed that with careful nutritional management, the KD could not be discontinued due to adverse reactions in the majority of patients.

The retention rate of 70.2% at 3 months, 43.2% at 6 months, and 25.7% at 12 months is a strong indicator of the overall tolerability of the KD and perceived benefit by caregivers. With increasing follow-up time, the retention rate of KD decreased gradually, particularly at 3 months. The primary reasons for KD discontinuation were a combination of lack of efficacy and intolerable adverse effects (28, 29). A large-scale cohort study indicated that anti-seizure efficacy played a predominant role in long-term retention, and only a few patients discontinued the KD due to adverse reactions (29). Therefore, we conducted survival analysis and showed that the retention rate in responders was better than that in non-responders at any point during the 12-month follow-up. This retention rate is notably encouraging given the challenges of maintaining a strict KD in infants, which involves meticulous preparation of specialized formulas (e.g., ketogenic breast milk fortifiers or formula) and the gradual transition to solid foods. The success is largely attributable to the intensive support provided by our dedicated team of neurologists, dietitians, and nurses, emphasizing that the KD is not merely a dietary prescription but a comprehensive therapy requiring a robust institutional support system.

This study has several limitations that should be considered when interpreting the findings. First, its single-center design may introduce selection bias and could limit the generalizability of the results to broader populations. Second, while the sample size is reasonable given the rarity of IESS, it remains relatively modest and may reduce the statistical power for conducting more nuanced subgroup analyses, such as comparing outcomes across specific etiologies or seizure types, potentially obscuring meaningful clinical distinctions. Third, while the duration of follow-up extended to 12 months, it remains limited for fully assessing the long-term sustainability, safety, and developmental impact of KD therapy. Finally, the study primarily focused on seizure control as a primary outcome and did not systematically evaluate long-term neurodevelopmental trajectories, which represent a critical endpoint in the management of IESS. Future larger multicenter and longer follow-up cohort studies with standardized developmental assessments are needed to validate these findings, optimize KD initiation and maintenance protocols, and identify reliable biomarkers that can predict treatment response.

## Conclusion

Our findings confirm that the KD represents an effective and well-tolerated therapeutic option for patients with IESS, particularly those unresponsive to standard pharmacological treatments. Although the successful implementation of this treatment demands diligent monitoring for adverse effects and collaborative effort between healthcare providers and families, the potential benefits are considerable. Future studies should prioritize the identification of reliable predictors of treatment response, refinement of initiation and maintenance protocols, and systematic evaluation of long-term neurodevelopmental and cognitive outcomes in this vulnerable population.

## Data Availability

The raw data supporting the conclusions of this article will be made available by the authors, without undue reservation.
